# The protective effect of hydromorphone to ischemia in rat glial cells

**DOI:** 10.1186/s40064-016-2281-7

**Published:** 2016-05-12

**Authors:** Young Sung Kim, Woon Young Kim, Yeon-hwa Kim, Ji Won Yoo, Too Jae Min

**Affiliations:** Department of Anesthesiology, Korea University College of Medicine, Seoul, Korea; Department of Anesthesiology and Pain Medicine, Korea University Ansan Hospital, 123, Jeokgeum-ro, Danwon-gu, Ansan-si, Gyeonggi-do 15355 Korea; Institute of Medical Science, Korea University Ansan Hospital, Korea University College of Medicine, Ansan, Korea; Department of Internal Medicine and Institute of Gerontology, University of Nevada, School of Medicine, Las Vegas, NV USA

**Keywords:** Hydromorphone, Neuroglia, Reactive oxygen species

## Abstract

**Background:**

Ischemic insults during operation can cause ischemic-reperfusion injuries in brain as well as memory impairments. Total intravenous anesthesia (TIVA) is the preferred anesthetic method in brain surgery, as it utilizes motor evoked potential monitoring. And the use of opioids is common in TIVA. However there are few studies about ischemic protective effect of opioids to glial cells.

**Methods:**

We used mixed cultures of rat glial cells, which were harvested from the brain of 1-day old rat. We divided the experimental groups according to their hydromorphone conditioning period: (a) pre-culture, (b) per-culture, or (c) pre- and per-culture. We measured the levels of the reactive oxygen species (ROS) induced by tert-butyl hydroperoxide (TBH) using flow cytometry. The ROS levels in the glial cells were also measured after the administration of 100 nM hydromorphone and selective opioid receptor antagonists.

**Results:**

The ROS levels were reduced in the hydromorphone-treated group, as compared to the control group (only TBH treated). There were no differences between pre-conditioned and per-conditioned groups. However, the ROS levels were more reduced in pre- and per-conditioned group compared to pre-conditioned or per-conditioned only groups. Furthermore, selective antagonists for the delta, kappa, or mu opioid receptor partially negated the hydromorphone effect.

**Conclusion:**

This study demonstrated that hydromorphone can have additional protective effects on oxidative stress when pre- and per-conditioning is combined. Furthermore we proved that μ, δ, κ opioid receptors participate in protective mechanism of hydromorphone to glial cells.

## Background

In recent times, glial cells are considered to represent a specialized functional unit with neurons, in addition to providing neuronal support. Glial dysfunction can cause cognitive disorders as well as short-term memory loss (Vicente et al. 2009; Maragakis and Rothstein 2006). Ischemic shock during surgery can induce ischemic-reperfusion injuries in the brain and memory impairments (Bilotta et al. 2013). Many studies have established that opioids are protective against ischemia and reperfusion in different cell types (Gross et al. 2010; Goldsmith et al. 2013). However, there are few studies that evaluate the protective effects of opioids in glial cells under ischemic conditions. Although opioids induce preconditioning effects in glial cells in the brain (Gwak et al. 2010), it is unknown whether opioids also have perconditioning effects.

Currently, total intravenous anesthesia (TIVA) is the preferred technique of anesthesia during neurosurgery due to its use of motor evoked potential (MEP) monitoring (Scheufler and Zentner 2002). The perioperative use of opioids is common in such settings. Therefore, it is meaningful to investigate whether opioids possess pre- and per-conditioning effects on glial cells. In this study, we evaluated whether hydromorphone had pre- and per-conditioning effect on glial cells. Furthermore we sought to determine whether hydromorphone had additive effects when pre- and per-conditioning were combined.

## Methods

### Chemicals

Hydromorphone was purchased from Hanapharm Co. (Seoul, Korea). Tert-butyl hydroperoxide (TBH, t-BOOH) and 2′,7′-dichloroflurorescin diacetate (DCF-DA) were purchased from Sigma Chemical Co. (St Louis, MO, USA). The anti-glial fibrillary acidic protein (GFAP) primary antibody and Alexa Fluor 488 goat anti-mouse antibody were purchased from Millipore Co. (Billerica, MA, USA) and Invitrogen Co. (Carlsbad, CA, USA), respectively. XTT was purchased from WELLGNE Inco. (Dalseo-gu, Daegu, Republic of Korea). The mounting solution was purchased from Vector Laboratories (Burlingame, CA, USA).

### Mixed primary rat glial cell culture

Rat glial cells were harvested and purified by the methods of previous study (de Vellis and Cole 2012). Pregnant Sprageue Dawley rat were purchased from Orientbio Co. (Seongnam-si, Gyeonggi-do, Republic of Korea). The rat cerebral cortices were purified from the brains of 1-day old rat pups, and then cultured in Dulbecco’s modified Eagle’s medium (DMEM) media supplemented with 10 % fetal bovine serum, 1 % antibiotics at 37 °C, and 5 % CO_2_ for 2 weeks. After 2 weeks, neurons, meningeal cells and fibroblasts were diminished, meanwhile microglial cells and astrocytes were replaced. We confirmed by immunostaining with anti-GFAP primary antibody via confocal microscope (Nikon A1si, Nikon, Japan). All procedures were reviewed and approved by the Committee on the Ethics of Animal Experiments of the Korea University Medical School. (IRB KUIACUC-2015-253)

### Cell viability

Cell viability was measured by XTT (2,3-bis-2-methoxy-4-nitro-5-sulfophenyl-2H- tetrazolium-5-carboxanilide inner salt, WelCount Cell Viability Assay kit). The mixed glial cells were cultured in a 96-well plate. The control, TBH (100 µM) and/or hydromorphone (100 nM) treated glial cells were placed in a new DMEM media, containing a 5 % XTT reaction mixture (50:1 = XTT solution: PMS solution) and incubated in a CO_2_ incubator, at a temperature of 37 °C for a period of 2 h. The XTT assay generated formazan crystals, which were measured spectro-photometrically with an ELISA reader (690 nm).

### Experimental protocol

**Fig. 1:**
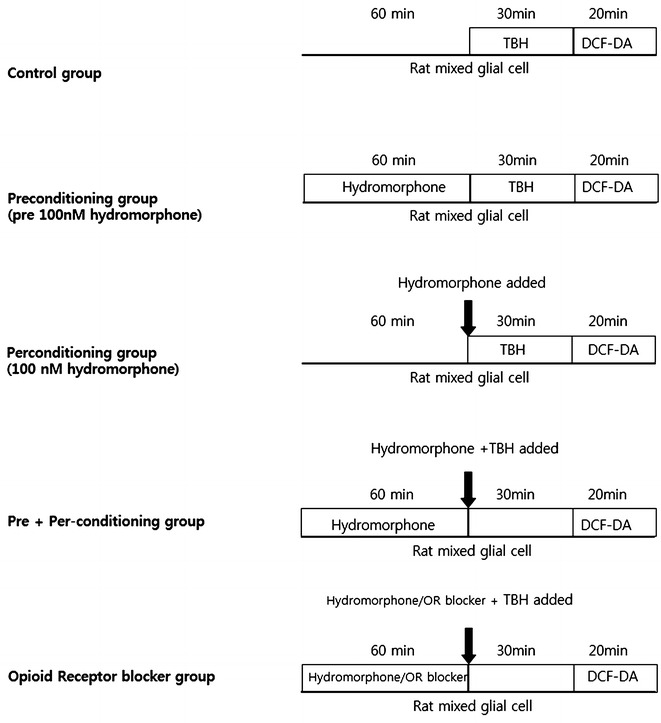
Experimental design. TBH: tert-butyl hydroperoxide, DCF-DA: 2′,7′-dichloroflurorescin diacetate, OR antagonist : naltrindole (a selective delta opioid receptor antagonist) or nor-binaltorphimine (a selective kappa opioid receptor antagonist) or naloxone

**Fig. 2:**
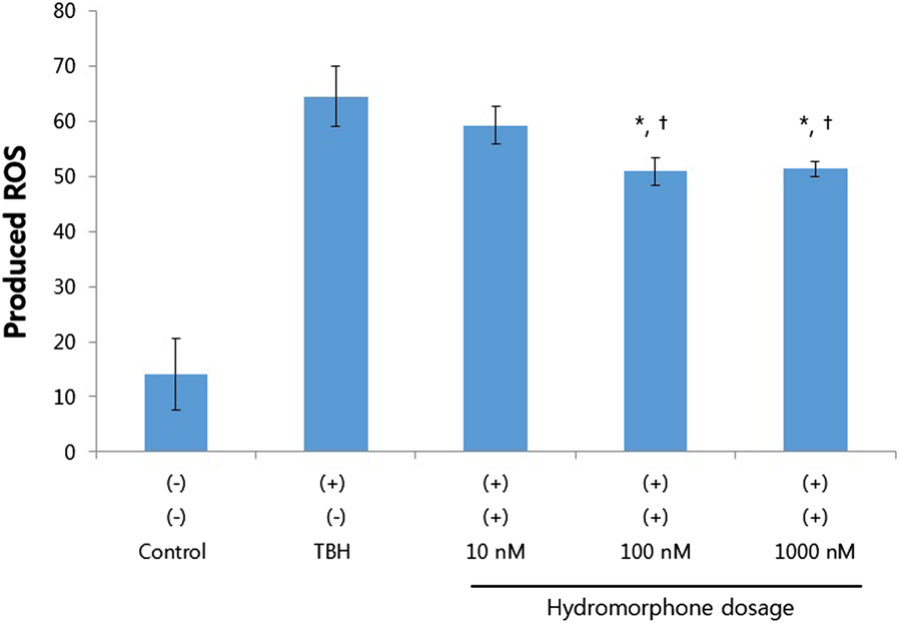
This graph shows effective dosage of hydromorphone for reducing ROS. The amount of ROS was expressed as Mean Fluorescence index. *<0.05 versus TBH group. ^†^<0.05 versus 10 nM hydromorphone group

The production of ROS in mixed glial cells was induced by TBH, which is organic peroxide (Peters et al. 2001), and then ROS was measured by flow cytometry using DCF-DA. The glial cells were proliferated to 5 × 10^4^ cells in 6-well plates, and then transferred to a serum-free media, which was prepared by overnight serum starvation. The cells were divided into 4 experimental groups : (1) pre-culture period (administration of 100 nM hydromorphone for 1 h, and then the media was changed to a serum-free media prior to 100 µM of TBH administration.); (2) per-culture period (100 nM of hydromorphone and 100 µM of TBH were co-treated.); (3) pre- and per-culture (100 nM of hydromorphone was pretreated for 1 h, and then the media was changed to a serum-free media. In addition, 100 µM of TBH and 100 nM of hydromorphone co-administrated for 30 min.); and (4) opioid receptor antagonist groups (pretreatment of 100 nM hydromorphone for 1 h, along with a selective delta- (δ-), kappa- (κ-), or mu-(μ-) opioid receptor antagonist, then cells were co-treated with TBH, opioid receptor antagonist, and 100 nM of hydromorphone) (Fig. 1). The dosage of opioid receptor antagonists were determined by previous study (Min et al. 2011). Also, Pilot study for determining the dosage of hydromorphone was performed. Three different doses (10, 100, 1000 nM) of hydromorphone were administrated. Each dosage of hydromorphone was treated for 1 h before 100 µM of TBH treatment (Fig. 2).

### The measurement of the intracellular reactive oxygen species (ROS) production

#### Flow cytometry

The control-, TBH- (100 µM) and/or hydromorphone- (100 nM) treated glial cells were incubated in a CO_2_ incubator with DCF-DA (10 µg/ml) at 37 °C for 20 min. The DCF-DA-treated glial cells were washed with phosphate-buffered saline (PBS) and detached from the plate using trypsin–EDTA (0.25 %). The detached cells were diluted with PBS (300 µl) for analysis by flow cytometry. At least three different sets of experiments were performed using cells from different isolations.

### TUNEL assay for apoptosis detection in glial cells

Apoptosis is characterized by DNA fragmentation, which can be detected by selective staining. A terminal deoxynucleotidyltransferase-mediated dUTP nick end labeling (TUNEL) apopto-sis staining kit (Roche Molecular Biochemicals, Mannheim, Ger-many) was used to detect programmed cell death. The glial cells were treated with hydromorphone (100 nM) for the pre-/per-period with TBH (100 µM) for 30 min), followed by fixation and TUNEL staining. The fixed cells were washed 3 times with PBS containing 0.1 %-Tween 20. The en-zyme and label solutions in the TUNEL stain kit were thoroughly mixed and reacted at 37 °C for 1 h. After the re-action, the cells were washed 3 times with PBS containing 0.1 %-Tween 20 and then examined using a confocal micro-scope. Three slides were examined for each group, and then particles were counted from 10 different parts of each slide.

### Statistical analysis

We used SPSS (Windows version 10.0, Chicago, IL, USA) for statistical analysis. All results are expressed as a mean ± standard deviation (SD) and are representative of three different experiments. The flow cytometry results were evaluated by one-way Anova. *P* values greater than 0.05 were considered significant.

## Results

### Cell viability

The cell survival rate after TBH was 84 %. This was calculated as a mean value.

### Hydromorphone dosage

The cells that were treated with 100 or 1000 nM of hydromorphone had significantly lower levels of ROS compared to those in the TBH-treated group (Fig. 2). There were no differences between ROS levels in the 100 and 1000 nM groups.

### The effects of TBH and/or hydromorphone on reactive oxygen species (ROS) production

**Fig. 3:**
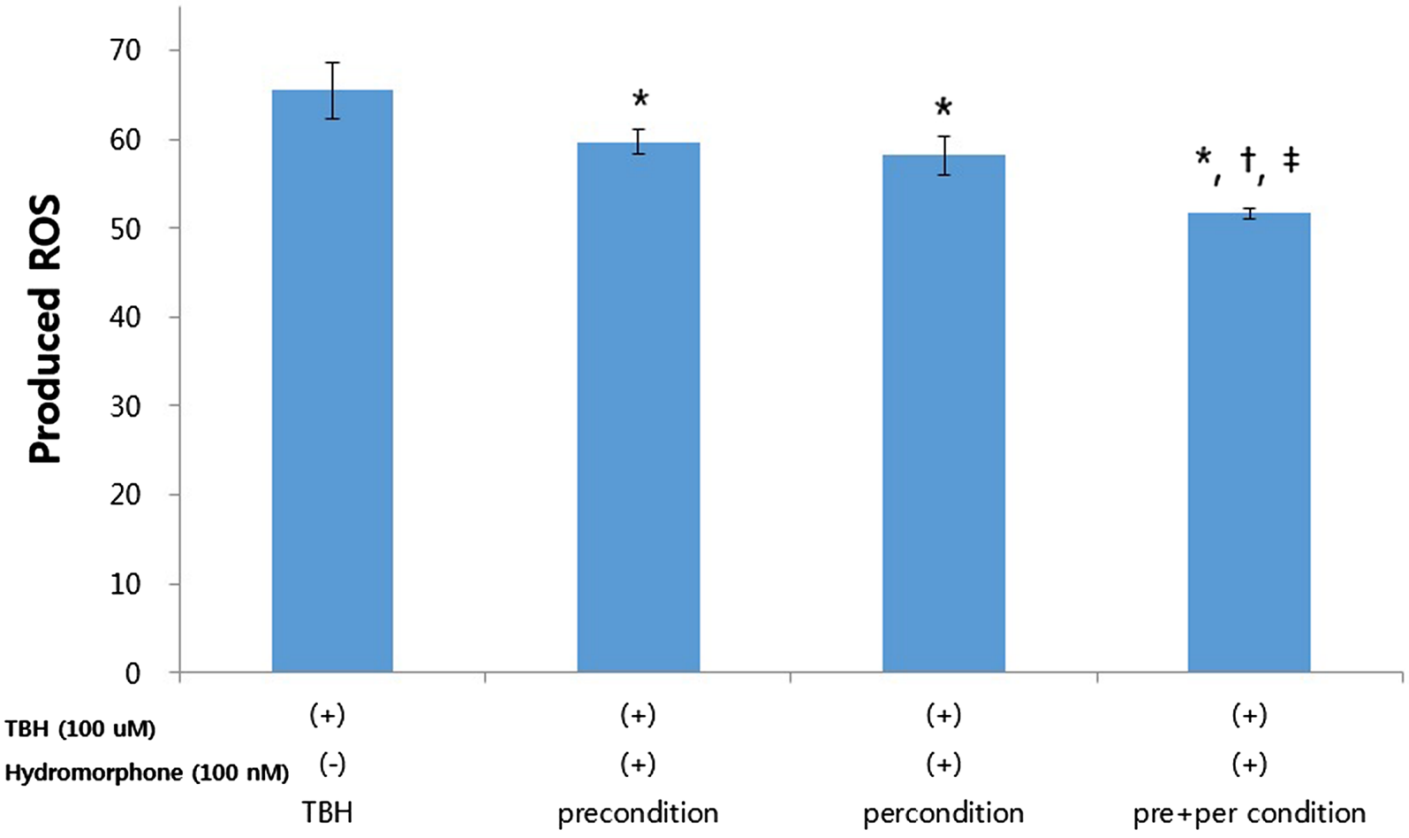
Detection of intracellular ROS (reactive oxygen species) product. This graph shows the protective effect of hydromorphone on TBH induced toxicity in primary rat glial cell cultures. The ROS level was measured with using FACS (DCF-DA) in the 100 nM hydromorphone (1) pre conditioned (2) per-conditioned (3) pre- and per- conditioned primary rat glial cells. The amount of ROS was expressed as Mean Fluorescence index. *<0.05 versus TBH group. ^†^<0.05 versus preconditioning group. ^‡^<0.05 versus perconditioning group

The ROS production was lower in the hydromorphone-treated group compared to the control group treated with TBH only. Moreover, the ROS production was significantly lower in the pre- and per-conditioned groups compared to the pre-conditioned or the per-conditioned alone groups (Fig. 3).

### The effect of opioid antagonists on reactive oxygen species (ROS) production

**Fig. 4:**
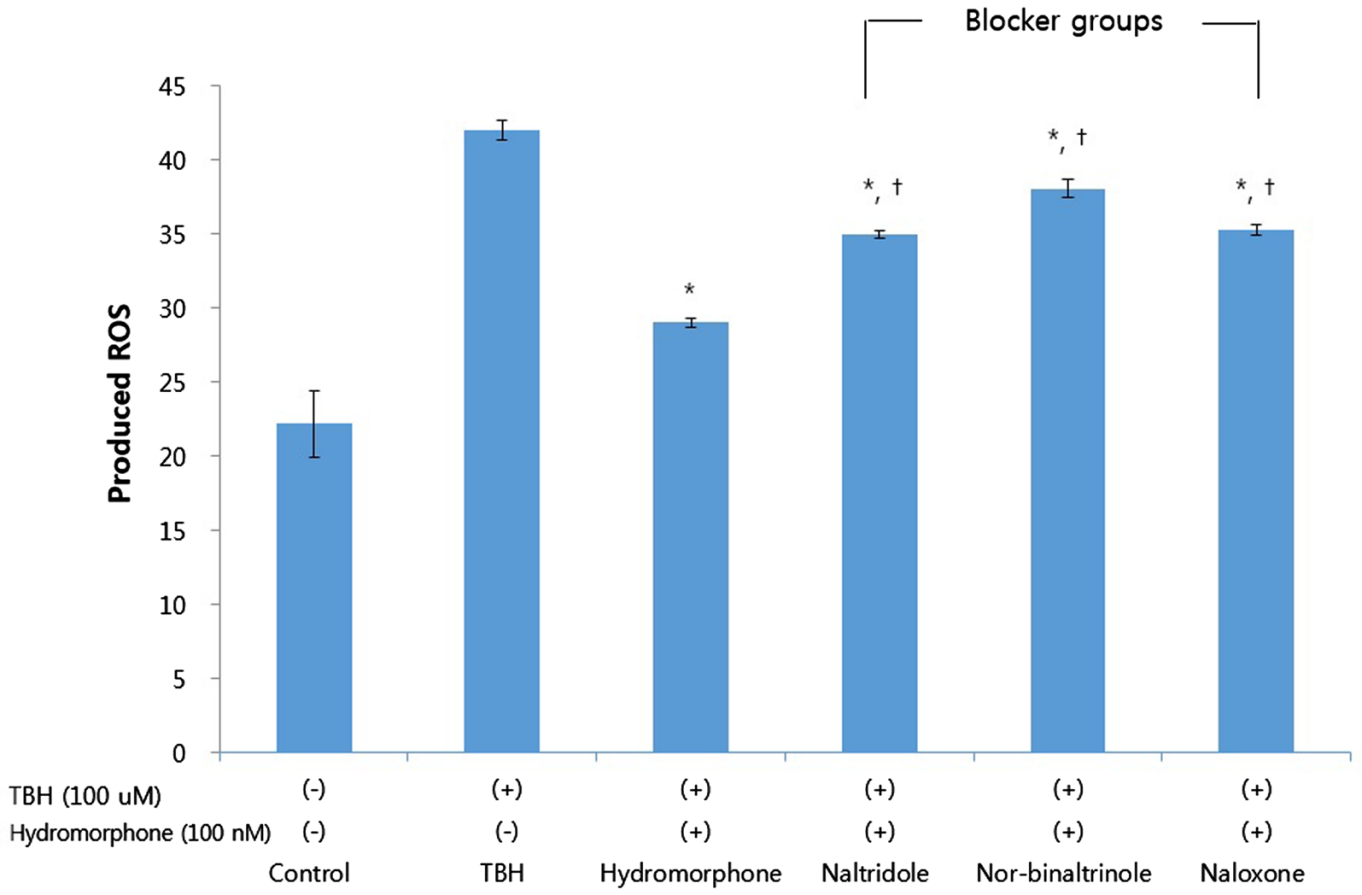
Effects of opioid receptor (OR) antagonists on reactive oxygen species (ROS) products. This graph shows that selective OR antagonists reverse the attenuation of the ROS induced by hydromorphone (pre and per-condition). The ROS level was measured with using FACS (DCF-DA) in the cells treated only TBH (control group), and the cells treated with TBH, hydromorphone and/or one of selective delta, kappa, and mu opioid receptor antagonists, naltrindole, nor-binaltorphimine, and naloxone, respectively. The amount of ROS was expressed as Mean Fluorescence index.*<0.05 versus control group. ^+^<0.05 versus hydromorphone group

The production of ROS was reduced in the hydromorphone-treated group. However, each of the selective δ-, κ-, and μ-opioid receptor antagonists partially negated the hydromorphone effects on ROS production (Fig. 4).

### TUNEL assay in glial cells

**Fig. 5:**
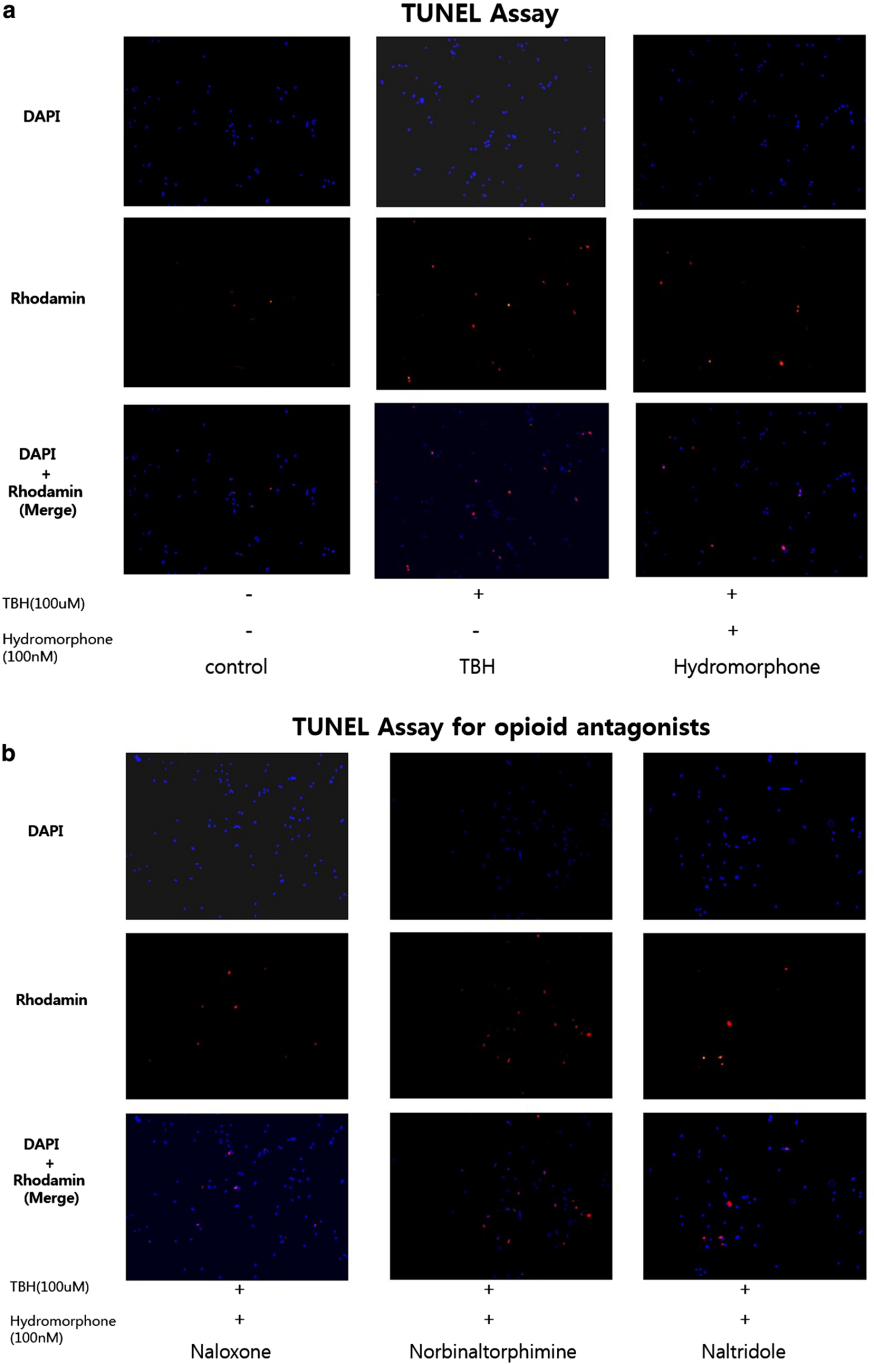
Terminal deoxynucleotidyl transferase-mediated dUTP nick end labeling (TUNEL) assay of rat glial cells. **a** Fluorescence micrographs of cells were stained with rhodamine and DAPI. Red particles represent DNA fragments. This graphs show the Control, TBH, and Hydromorphone groups. **b** Fluorescence micrographs of cells were stained with rhodamine and DAPI. Red particles represent DNA fragments. This graphs show the opioid antagonist groups. ^†^<0.05 versus hydromorphone group

**Fig. 6:**
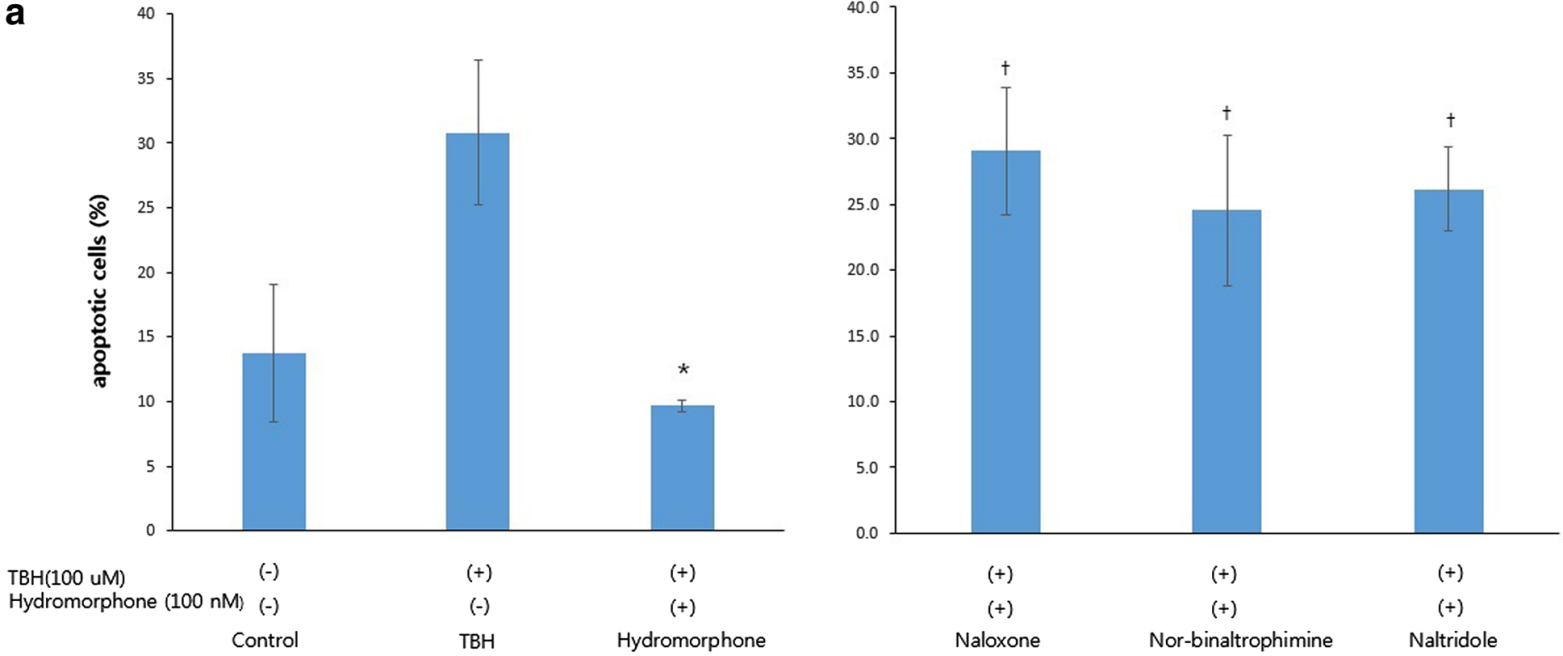
Terminal deoxynucleotidyl transferase-mediated dUTP nick end labeling (TUNEL) assay of rat glial cells. **a** This graph shows quantitation of apoptotic cells from TUNEL assay by number of red particles. *<0.05 versus TBH group. ^†^<0.05 versus hydromorphone group

The apoptotic cells appeared as light red dots under a fluorescent microscope after performing the TUNEL as-say. A comparison between the TBH group and the pre- and per-conditioned group revealed that there were significantly less apoptotic cells in the pre- and per-conditioned group. Furthermore, the number of apoptotic cells in the opioid receptor blocker group was significantly increased (Figs. 5, 6).

## Discussion

Currently, TIVA is the preferred method for general anesthesia during cerebral surgery because TIVA barely affects MEP monitoring (Zhang et al. 2011, 2004). Opioid use for intra-operative analgesia is common in TIVA. In this study, we demonstrated the pre-conditioning and per-conditioning effects of hydromorphone, providing evidence for the peri-operative effectiveness of hydromorphone.

Recent studies have shown that opioids, including morphine, have protective preconditioning effects in several ischemia/reperfusion models (Zhang et al. 2011, 2004). Morphine has anti-neuroinflammatory properties and can prevent memory deficits due to its preconditioning effect, which is dependent on opioid receptors (Rostami et al. 2012). However, the role of opioids in glial cells is unclear. Although glial cells play an important role in the antioxidant defense system in the brain (Iwata-Ichikawa et al. 1999), they are considered to be functional units rather than the barriers of neurons. Glial cell dysfunction can cause cognitive impairments and short-term memory loss (Vicente et al. 2009; Maragakis and Rothstein 2006). In addition, neuroinflammation is highly associated with ischemic stroke-induced cerebral damage and the progression of neurodegenerative diseases (Mucke and Eddleston 1993; Kreutzberg 1996; Brown and Bal-Price 2003). Glial cells are important because they are responsible for the brain’s neurophysiology and hence, represent potential therapeutic targets.

Excessive oxidative stress produces excessive ROS. These ROS play important roles to CNS damage during ischemic conditions (Kontos 1989) and are associated with a variety of diseases (Hu et al. 2015). Although a certain amount of ROS is essential for cellular survival, cell death can occur when ROS-induced oxidative stress exceeds a cell’s antioxidant capabilities (Hu et al. 2015; Yan 2014). Excessive ROS production can cause DNA damage, modify protein and lipid functions, and activate signal pathways (Yan 2014). However, a moderate level of oxidative stress, referred to as positive oxidative stress, can be induced and modulated to produce an adaptive cellular response that is beneficial for cellular survival. Both preconditioning and per-conditioning effects can potentially be the result of positive oxidative stress (Yan 2014; Milisav et al. 2012). Brief episodes of ischemia and reperfusion, and a number of pharmacological agents, can induce these phenomena (Das and Das 2008). TBH is commonly used to induce oxidative stress in vitro and in vivo (Rush et al. 1985). In this study, we used TBH to induce oxidative stress-related injury to rodent-derived glial cells (Holownia et al. 2009).

Several studies have demonstrated that morphine is involved in immune system functions (Hutchinson et al. 2007; Madera-Salcedo et al. 2011). Morphine exhibits both peripheral anti-inflammatory and inflammatory roles (Askari et al. 2008; Pourpak et al. 2004). Although opioids can induce glial activation and neuroinflammatory reactions (Watkins et al. 2009), morphine-induced preconditioning has been found to be anti-neuroinflammatory (Rostami et al. 2012). In these studies, we used hydromorphone, an opioid receptor agonist and morphine derivative (Ricket et al. 2015), to prevent TBH-induced ROS production. Hydromorphone, one of the first line opioids as per the European guideline (Oosten et al. 2015), is widely used for acute and chronic pain management in both adults and children (Oosten et al. 2015; Mherekumombe and Collins 2015). Compared to morphine, hydromorphone has certain clinical advantages, such as reduced nausea and vomiting (Wirz et al. 2008), and lower histamine release (Guedes et al. 2007). To demonstrate its protective effect, we used hydromorphone to induce oxidative stress-related injury. Because there are few in vitro studies using hydromorphone, we choose an equi-analgesic dosage of hydromorphone as compared to morphine, based on previous studies (Min et al. 2011; Lee et al. 2012). Pilot studies determined the maximum effective dose of hydromorphone for a pre-/per-conditioning effect, which was 100 nM (Fig. 1).

In this study, hydromorphone was protective against TBH-induced oxidative stress-related injury. Interestingly, the combined administration of hydromorphone (pre-conditioning followed by co-administration) had a superior effect compared to pre-conditioning and co-administration treatments alone. Opioids can not only suppress responses to various noxious stimuli (e.g., short cyclic episodes of ischemia or ROS), but also have similar pre-conditioning effects (Rostami et al. 2012; Das and Das 2008). This cumulative effect of hydromorphone may be important for clinical settings, such as the perioperative use of hydromorphone in neurosurgery.

The endogenous opioids and their receptors (μ-, δ-, κ-) are widely distributed in the glials like astrocytes (Pearce et al. 1985), oligodendrocytes (Knapp et al. 1998) and microglial cells (Calvo et al. 2000; Turchan-Cholewo et al. 2008). They exert their physiological effects, including nociception, analgesia, and brain development. Recently, Jeong et al. (2012) reported that remifentanil which is selective agonist for δ-opioid receptor, has neuroprotective effect to both neurons and glial cells, improves neurological scores, and reduces infarct volumes via the attenuation of mitogen-activated protein kinases (MAPK). MAPK involves ROS production and the release of pro-inflammatory cytokines (Son et al. 2011). But the protective effects of μ- and κ-opioid receptors to cerebral ischemia are still controversial. Some of studies have elucidated the protective function of κ-opioid receptors to reperfusion injury in ischemic cerebral animal models (Zhang et al. 2003; Goyagi et al. 2003). These effects are mediated through reducing NO production. Goyagi et al. (2003) showed that activation of κ-opioid receptors attenuates the evokation of NMDA by inhibiting excitatory postsynaptic potentials, and NMDA is involved in the NO production of brain. NMDA receptors are abundant in glial cells and neurons, and have important roles to maintain the homeostasis of brain (Verkhrataky and Kirchhoff 2007). These evoked *N*-Methyl-d-aspartic acid (NMDA) also induces ROS products (Shelat et al. 2008). Our results demonstrate that each opioid receptor subtype, the μ-, δ-, and κ-opioid receptor, reduces ROS production and is involved in the protective effects of hydromorphone in mixed glial cells. After administration of selective μ-, δ-, and κ-opioid receptor antagonists, ROS production was similar to that of the TBH-treated group, suggesting that the μ-, δ-, and κ-opioid receptors all contribute to hydromorphone’s protective effects. Our data also suggest that hydromorphone has pre and per conditioning effects as well. Considering the present results, MAPK pathways and NMDA signaling pathways might be involved in the protective effect of hydromorphone on TBH-induced glial cells. Further studies are required to evaluate the mechanism underlying opioid receptors involvement by using knockout models.

In conclusion, this study provides evidence that hydromorphone has both, pre-conditioning and per-conditioning effects on the TBH-induced injuries. Furthermore, pre- and per-conditioning effects of hydromorphone is additive. We also demonstrated that the μ-, δ-, and κ-opioid receptors all contributes to the mechanism of hydromorphone and its protective effect on glial cells. Hence, our findings support the therapeutic potential for hydromorphone in the prevention of ischemic cerebral injury during the perioperative period.

## Abbreviations

TBH: tert-butyl hydroperoxide
ROS: reactive oxygen species
TIVA: total intravenous anesthesia
MEP: motor evoked potential
